# The Characterisation of Pluripotent and Multipotent Stem Cells Using Fourier Transform Infrared Microspectroscopy

**DOI:** 10.3390/ijms140917453

**Published:** 2013-08-26

**Authors:** Julie Cao, Elizabeth S. Ng, Donald McNaughton, Edouard G. Stanley, Andrew G. Elefanty, Mark J. Tobin, Philip Heraud

**Affiliations:** 1 Department of Anatomy and Developmental Biology, Faculty of Medicine, Nursing and Health Sciences, Monash University, Wellington Road, Clayton, Victoria 3800, Australia; E-Mails: julie.cao@monash.edu (J.C.); elizabeth.ng@monash.edu (E.S.N.); ed.stanley@monash.edu (E.G.S.); andrew.elefanty@monash.edu (A.G.E.); 2 Centre for Biospectroscopy and the School of Chemistry, Monash University, Clayton, Victoria 3800, Australia; E-Mail: don.mcnaughton@monash.edu; 3 Murdoch Childrens Research Institute, the Royal Children’s Hospital, Parkville, Victoria 3052, Australia; 4 Australian Synchrotron, 800 Blackburn Road, Clayton, Victoria 3168, Australia; E-Mail: mark.tobin@synchrotron.org.au

**Keywords:** Fourier transform infrared microspectroscopy, pluripotent stem cells, multipotent stem cells, regenerative medicine

## Abstract

Fourier transform infrared (FTIR) microspectroscopy shows potential as a benign, objective and rapid tool to screen pluripotent and multipotent stem cells for clinical use. It offers a new experimental approach that provides a holistic measurement of macromolecular composition such that a signature representing the internal cellular phenotype is obtained. The use of this technique therefore contributes information that is complementary to that acquired by conventional genetic and immunohistochemical methods.

## 1. Introduction

Owing to their unique potential to differentiate into the three embryonic germ layers and give rise to all the cell types of the embryo proper, pluripotent stem cells hold much promise in the field of regenerative medicine. They include embryonic stem cells [1], so called because of their derivation from blastocyst stage embryos, and induced pluripotent stem cells [2], aptly named because pluripotency has been “induced” via the reprogramming of their somatic cell progenitors. In contrast, adult stem cells are mostly “multipotent”, which means that they are able to give rise to a subset of cell lineages, and of these, the hematopoietic stem cells are the best characterised population [3].

**Figure 1:**
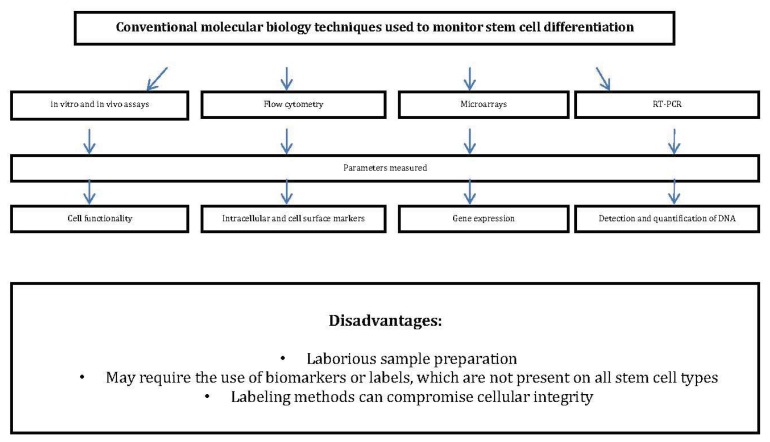
Flow chart summarising conventional molecular biology techniques currently used to monitor stem cell differentiation, the parameters that they measure, and their disadvantages.

Before the envisaged clinical applications, numerous challenges pertaining to the isolation, identification, enrichment and purification of differentiated stem cells must be overcome [4–6]. The development of more robust screening processes is critical because the inadvertent transplantation of populations of undifferentiated stem cells has been known to lead to the formation of non-malignant tumours called teratomas. At present, stem cell differentiation is monitored via the use of a number of molecular biological techniques, which include *in vitro* and *in vivo* assays, flow cytometry, real time polymerase chain reaction (RT-PCR) and microarray technologies [7] (Figure 1). These techniques not only require time consuming sample preparation, but also involve the use of biomarkers or labels, which are absent on certain cell types such as cardiomyocytes, gastrointestinal stem cells [8], and corneal stem cells [9]. Furthermore, these label driven methods have been known to reduce sample integrity by causing cellular stress and damage, thereby affecting the cells’ behaviour. Given the insufficiencies of these methods, there is a clear need amongst stem cell biologists, to implement an objective, label-free, non-destructive technique for the screening of stem cells and their derivatives.

The recent adoption of vibrational spectroscopic approaches to study stem cell differentiation has emerged as a feasible solution to this problem [10]. One of these modalities, Fourier transform infrared (FTIR) microspectroscopy, has been the subject of preliminary studies by various groups to interrogate both pluripotent and multipotent cells. Whilst the study of biological samples using FTIR microspectroscopy has been successful for more than half a century [11,12] laying the foundation for our current understanding of their IR band assignments, its application to stem cells has only taken place within the last few years.

## 2. FTIR Microspectroscopy—A Concise Background

Mid infrared FTIR spectroscopy, based on radiation absorption between 2.5 μm and 25 μm wavelengths (4000–400 cm^−1^) exploits the intrinsic property of molecular systems to vibrate in resonance with different frequencies of infrared light. In biological samples, the vibrational modes in macromolecular molecules, such as proteins, lipids, carbohydrates and nucleic acids, give rise to a series of clearly identifiable functional group bands in FTIR spectra, providing us with information about relative concentrations and specific chemical structures [13]. Band assignments of mid-IR spectra common to biological samples are presented in Table 1 according to foundation publications in the literature.

The most prominent band in biological spectra is the amide I band which arises from the coupled C=O stretching and N–H bending of proteins [14,15] and is sensitive to changes in protein secondary structure. However, the interpretation of protein information in this region of the spectrum needs to be made with caution, since contamination of the spectra from water vapour and distortion through light scattering can cause profound changes in this spectral region. Recent approaches that have been developed to mitigate these effects are claimed to be successful [16,17], but further studies are required to assess their true efficacy.

### FTIR Microspectroscopy Instrumentation

The types of FTIR instruments that have been used to interrogate stem cells include benchtop FTIR microspectroscopic instruments which utilise either globar or synchrotron mid-infrared radiation sources. Recently, the coupling of focal plane array (FPA) detectors to FTIR microscopes has allowed for the acquisition of thousands of spectra in a single experiment [18]. Nevertheless, despite the speed advantages, such systems achieve this at the expense of decreased spatial resolutions and signal to noise ratios when compared to synchrotron based experiments which utilise single detectors. An exception to the latter is the multi-beam IR synchrotron source at the IRENI (IR Environmental Imaging) beam line located within the Synchrotron Radiation Centre (SRC) (Madison, WI, USA) [19] which employs an FPA detector.

To achieve diffraction limited spatial resolution with single point detectors, the infrared beam size is confined to a smaller region of interest via decreasing the aperture in the IR microscope. However, doing this with a Globar IR source results in degraded signal to noise ratios due to an insufficient amount of IR light reaching the detector. By using the highly brilliant, collimated beam of photons generated by synchrotron IR sources, spectra can be acquired using an aperture of 5 μm × 5 μm because the SNR can be ~1000 times higher than can be achieved with globar IR sources [20]. It should be noted however, that due to the effects of diffraction, the obtainable spatial resolution is a function of wavenumber, and is not necessarily defined by the microscopic aperture that is employed.

More recently, advances in IR instrumentation have seen the utilisation of quantum cascade laser (QCL) sources of mid infrared light [21], which are more brilliant than synchrotron sources and allow for FTIR measurements with high signal to noise ratios to be achieved in the laboratory, even with single living cells. However, a few major challenges need to be overcome before they can be widely adopted [22]. The first one is concerned with how to remove the mid-IR laser speckle, which arises from the use of coherent illumination, and significantly reduces image resolution. The second major issue relates to determining the optimal way to couple the laser radiation into the microscope, so that the former issue, in addition to others, can be fully investigated. Thirdly, QCL have restricted tuning ranges and because they require scanning, data acquisition is considerably slower. As a consequence, the broadband, multiplexing nature of FTIR is lost.

## 3. Interpreting Features of the FTIR Spectrum

Since the identification of most spectral features is difficult in the raw spectrum due to bands from multiple vibrational modes being superimposed, it is a common practice to convert biological IR spectra to their derivatives, often the second derivative [23], apply a de-noising filter such as smoothing, and correct for path length differences using normalisation techniques such as vector normalisation, or more powerful methods such as multiplicative scatter correction (MSC) or extended multiplicative signal correction (EMSC) [24]. The resultant spectra possess a flatter baseline, and for second derivative spectra, negative peaks, which are directly aligned to the centre of the underivatised spectrum’s absorbance peak, enabling previously overlapping bands to be resolved. Whilst comparing the average second derivative spectra can be useful in discerning differences in band intensities between different spectral datasets, it can be misleading especially when dealing with a small number of spectra, or if there are different levels of heterogeneity in the sample dataset. Accordingly, spectroscopists tend to employ more objective, multivariate approaches for the analysis of spectral trends. Typical methods used in these studies include Principal Component Analysis (PCA), Linear Discriminant Analysis (LDA), Unsupervised Hierarchical Cluster Analysis (UHCA) and Artificial Neural Networks (ANNs).

### 3.1. Principal Component Analysis (PCA)

**Figure 2:**
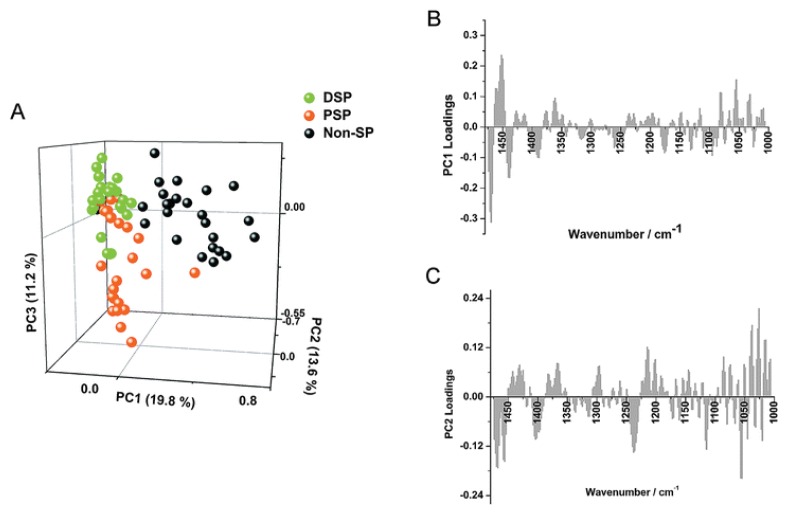
PCA of subsets of “stem cell-like” renal epithelium carcinoma cells. Included in the analysis were the sub-side populations, distal side population (DSP) and proximal side population (PSP) *vs.* non-side population (Non-SP) cell spectra. (**A**) The scores plot of PC1, PC2 and PC3 and (**B**) corresponding loadings of PC1 and (**C**) PC2 are shown. Key biochemical differences are outlined in lipid, phosphodiester and carbohydrate absorption bands [26].

Owing to the intrinsic multi-dimensionality of IR spectra, the task of interpreting trends within the dataset can be difficult. PCA allows for the reduction in the dimensionality of spectral datasets, thereby facilitating the identification of any clustering patterns that may exist [25]. In PCA, each spectrum is represented as a single point on a scores plot with axes called principal components (PCs). These PCs comprise a new set of variables, maintaining as much of the original variation as possible, with the first PC representing the vector showing the direction of greatest variation in the spectral dataset and subsequent PCs showing the direction of next greatest variation in the dataset, with these sources of variation independent to previous PCs. This condensing of the larger number of original variables to a smaller number of variables allows for the most relevant analytical information to be presented. In PCA, a loadings plot or “pseudo spectrum” is calculated for each PC, which displays the variance at each wavenumber in the spectral dataset (Figure 2).

### 3.2. Linear Discriminant Analysis (LDA)

**Figure 3:**
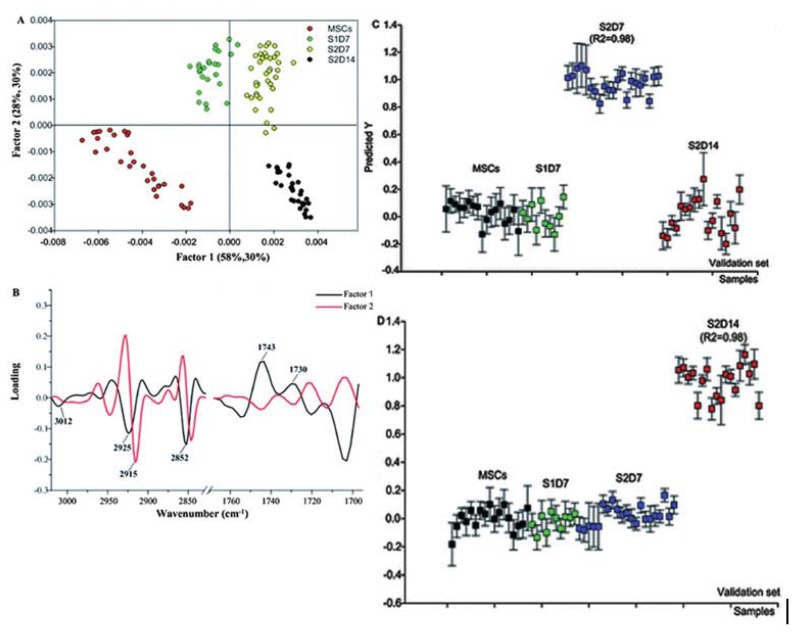
Scores and loadings plots from the PLS-DA of FTIR spectral data acquired at different stages of hepatic differentiation. (**A**) Scores plot showing factors 1 and 2, explaining 58% and 28% of the sample variance, respectively; (**B**) loading plot for factors 1 and 2 showing the most variable spectral regions explaining the PLS-DA. PLS-DA results of spectra drawn from the four investigated cell classes: undifferentiated rBM-MSCs, early stage cells (S1D7), mid-stage cells (S2D7) and late stage cells (S2D14) (**C**,**D**). The correlation coefficients (*R*^2^ > 0.98 for all calibration sets) indicated that all the datasets were well modelled. PLS-DA correctly classified and discriminated all of the validation spectra [27].

LDA is a factor analysis method which involves the decomposition of a matrix of spectra into matrices which consist of loading spectra and scores. The original spectra can be thought of as linear combinations of the loading spectra and the loadings’ contributions are denoted by the scores. This technique ensures that inter-class separation is maximised whereas any intra-class separation is minimised. Often, a cross-validation step is implemented, where the model is validated by using a supervised training dataset, followed by classification of an independent validation test set (Figure 3).

### 3.3. Partial Least Squares Discriminant Analysis (PLS-DA)

Partial Least Squares (PLS) analysis is another multivariate analysis technique that is used to decompose and identify structure in large datasets [28], and is often used in conjunction with LDA. PLS involves the identification of a set of components called latent vectors, which decomposes the *X* (predictor) and *Y* (dependent) matrices simultaneously and is followed by a regression step where the decomposition of *X* is used to predict Y. In Partial Least Squares Discriminant Analysis (PLS-DA) the calibration data matrix consists of the spectral dataset (multivariate *X*) and a *Y* matrix containing variables with integer values of 0 or 1 coding for the each of the modelled spectral classes. Classification of the dataset is then carried out by predicting a *Y* value for each spectrum in an independent validation using PLS models that had been generated from the calibration set. Correct classification of each class are arbitrarily assigned to samples with predicted *Y* > 0.5 for respective spectra.

### 3.4. Unsupervised Hierarchical Cluster Analysis (UHCA)

**Figure 4:**
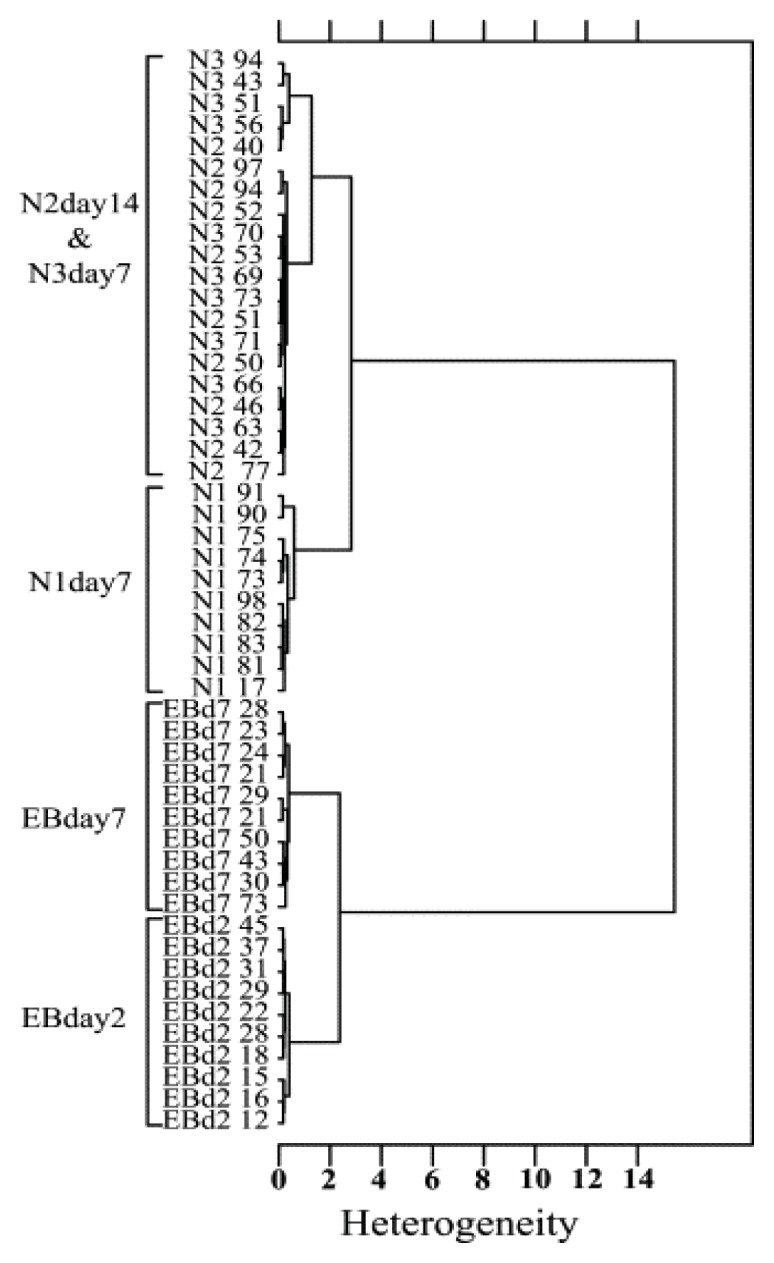
UHCA results of mouse embryonic stem cells and progenitor cells at different stages of neural differentiation. Cluster analysis employed Ward’s algorithm using second derivatives, vector normalised spectra, over the spectral ranges 3000–2800 cm^−1^ to 1750–900 cm^−1^ [29].

In Unsupervised Hierarchical Cluster Analysis (UHCA), spectral distances are measured using the pre-processed dataset to elucidate the degree of similarity between two spectra or clusters, with more similar spectra having smaller distances [29]. The clustering process itself is performed by an algorithm, such as Ward’s algorithm, which utilises a matrix defining inter-spectral distances to determine the two most similar IR spectra via combining the two clusters with the smallest degree of heterogeneity into a new cluster or hierarchical group (Figure 4).

### 3.5. Artificial Neural Networks

An artificial neural networks (ANN), is an example of a non-linear pattern recognition approach that can be used in FTIR microspectroscopy of stem cells, and is analogous to biological neural systems [30]. Their working environment consists of numerous interconnected processing elements (neurons) that work in cohesion to gain, predict, interpret and represent data in order to solve multifaceted user-specific problems. The architecture of an ANN consists of several “neuronal” layers, adjoined by “synapses”. Data are first sent to the input layer, which sends this via the synapses to the middle layers, and then to the output layer. For instance, in the case of FTIR data, the number of input neurons often equates to the number of individual wavenumber measurements in the spectrum, whereas the output neurons correspond to the different classification groupings. Parameters called “weights”, which manipulate the data in the calculations are stored in the synapses, and can be optimised during the training phase of the ANN via back propagation, which adjusts these, in addition to other biases via calculating the gradient of the error.

All of these approaches to spectral classification have been widely used in biospectroscopy in general, including stem cell spectroscopy work (pertinent examples are: PCA [31]; LDA [8]; UCHA [29]; and ANNs [32]).

## 4. FTIR Microspectroscopy Discriminates between Stem Cells of Different Potencies

The applicability of FTIR microspectroscopy for discriminating between stem cells with different intrinsic potencies based on their spectral “signatures” was demonstrated by one laboratory which compared the spectral signatures of individual pluripotent stem cells (hESC) from individual multipotent stem cells (hMSC) [31]. Examination of their IR spectra revealed differences in spectral regions associated with lipids. For instance, the hESC spectra displayed more intense peaks at 2920 cm^−1^ and 2850 cm^−1^ and at 1740 cm^−1^, which were red shifted by approximately 4 cm^−1^, relative to the single cell spectra of the hMSC. These observations were validated by lipid staining tests, which revealed higher lipid concentrations in the cytoplasm of the hESC compared with the hMSC. These data suggested a possible link between lipid concentrations and the potency of a stem cell, which might be related to the actions of the eicosanoid pathway, however, further work is required to verify this.

## 5. Pluripotent Stem Cell Applications—Embryonic Stem Cells

Various laboratories have demonstrated the utility of FTIR microspectroscopy for elucidating the biochemical differences that distinguish the spectra of murine and human embryonic stem cells from their lineage committed progeny. In the first published hESC study [32], our colleagues showed that the differences between spectra of hESC lines and their derived cell types could be detected after only four days of differentiation.

**Figure 5:**
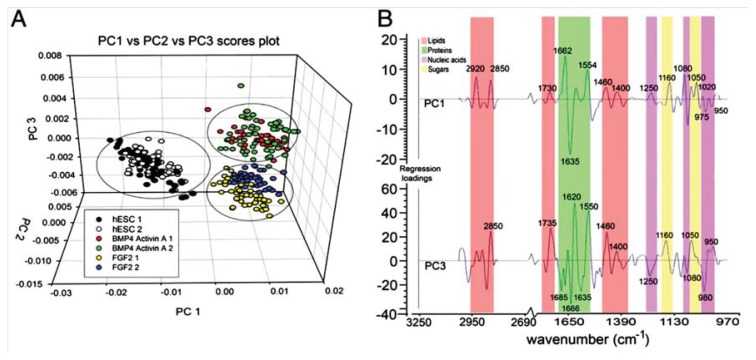
(**A**) Scores plots for PLS-DA of the spectral data for the three treatment groups from one experiment showing replicate samples for each cell type. Each spectrum is represented as a point in PC1 *vs.* PC2 *vs.* PC3 space; (**B**) Regression coefficient plots used to explain the clustering observed in the PLS scores plot shown in panel A. PC1 regression coefficient loadings indicated spectral differences explaining clustering along PC1, which separates hESCs from the cells differentiated in FGF2 or BMP4/Act A. PC3 regression coefficient loadings explained the separation of the spectra from FGF2-and BMP4/Act A-treated sample groups along PC3. Limited spread in the PC2 direction did not contribute significantly to the clustering of samples [32].

In this work, FPA-FTIR microspectroscopy was used to acquire spectra of hESC that were differentiated towards mesendoderm (precursor cells of mesoderm and endodermal embryonic germ layers) induced by a bone morphogenetic protein cytokine cocktail (BMP4/Act A medium) or towards ectodermal lineages (precursor cells of the skin and nervous system) in medium supplemented with fibroblast growth factor (FGF2) (Figure 5). Across all three experimental replicates, the undifferentiated human embryonic stem cell line, *MIXL1*^GFP/w^, could be discriminated from its differentiated progeny due to spectra of the undifferentiated cells possessing higher IR absorbance for lipid and glycogen components. The distinctness of the spectra of the progeny cells from the spectra of the stem cells was further confirmed using various multivariate data analysis approaches including PCA, PLS-DA and ANN modelling.

Others who have exploited this tool to study embryonic stem cell differentiation have utilised mouse derived cells. One of the first groups to do so employed FTIR microspectroscopy and PCA-LDA approaches to identify the unique marker bands of spontaneously differentiated murine embryonic stem cells that occurred during known biological stages of ES cell differentiation [33]. Multivariate analysis revealed an excellent separation of spectral clusters between the undifferentiated cells and cells that were differentiated at days 4, 7, 9, and 14, in the PCA-LDA score plots. The group successfully identified pronounced protein secondary structural changes, in the 1700–1600 cm^−1^ region between undifferentiated cells and their cardiomyocyte precursors. Additionally, they observed a marked increase in the expression of α-helical structures between day 0 and day 10 of differentiation, and noticed that the β-turn secondary structures were enriched, although the latter was not present in the undifferentiated cells. Further to these overall protein changes, the authors reported simultaneous intensity decreases at 994 cm^−1^ and at 914 cm^−1^, which were assigned to the nucleic acid absorption bands, up to 9 days after differentiation, suggestive of the occurrence of mRNA translation. Other observations were a decrease in the 966 cm^−1^ band intensity, assigned to the DNA C–C stretching of the backbone and RNA ribose phosphate main chain modes in undifferentiated cells. Between days 4 and 7 of differentiation, they believed that the active transcription of the genome was switched on, as evidenced by the appearance of new bands at 954 cm^−1^ and at 899 cm^−1^ (assigned to the C–C vibration of the A-DNA backbone and to the deoxyribose ring vibration respectively).

In another successful application of FTIR microspectroscopy to discriminate different types of differentiated mESCs, synchrotron FTIR microspectroscopy was implemented to study the differentiation of stem cells into hepatocyte-like cells [34]. The differentiation of mouse embryonic stem cells into hepatocyte-like cells is a complex, multistage process, which first consists of endoderm induction, followed by hepatic initiation, and finally, maturation into hepatocyte-like cells. Taking advantage of the high brilliance of the synchrotron infrared radiation, the group was able to accurately discriminate between the three differentiating cell populations at the single cell level.

For instance, mature hepatocyte-like cells were found to have heavy loadings at 1656 cm^−1^, assigned to α-helix protein secondary structures, when compared with spectra of progenitor cells, which were seen to cluster away from these groups along PC1. This finding was corroborated by the fact that mature hepatocyte like cells express proteins characteristic of mature albumin, which is rich in α-helical structures, and the α-feto-protein. Along PC2, a separation was observed between spectral clusters of progenitor cells from spectra of the mature hepatocyte cells, with the former having heavy loadings at 1627 cm^−1^, assigned to β-sheet secondary structure of proteins. Lipid differences could also explain the separation observed along this PC, with spectral clusters of hepatic progenitor cells having higher absorbance for loadings bands associated with C-H stretching regions at 2852 cm^−1^ and 2923 cm^−1^, and the ester carbonyl stretching mode at 1740 cm^−1^. Subsequent PLS modelling of the spectral datasets showed that the three differentiated cell types could be discriminated with very high sensitivity and specificity.

FPA and synchrotron-based FTIR microspectroscopy (SR-FTIR) has proved useful for discriminating mouse embryonic stem cells differentiated towards embryoid bodies (EBs), from neural progenitor cells (NPCs), and embryonic stem derived neural cells (ESNCS) [29]. The authors observed macromolecular chemical differences between the different differentiated cell types, mainly occurring in the spectral regions assigned to protein and lipid absorbance. During the differentiation of NPCs and ESNCs, protein secondary structural changes were observed, with increases in α-helical and decreases in β-sheet secondary structure. These findings were in agreement with existing non-spectroscopic data which have shown similar higher levels of α-helical structure proteins occurring with neural differentiation, possibly due to alterations in cytoskeleton proteins. Further, the differentiation of neural progenitors and mature neural cells were found to correspond to changes in the peak position and spectral intensity of the asymmetric and symmetric CH_2_ stretching modes, and ester carbonyl band ascribed to lipid components, thought to be due to increased expression of glycerophospholipids, which are involved in neural cell proliferation, differentiation, and signal transduction. Spectral differences from each stage of differentiation could be seen via interrogating spectra of both clumps, and individual cells, and the biochemical entities explaining the differences between the EBs and the ESNCs were verified using PCA and unsupervised hierarchical cluster analysis (UHCA).

## 6. Pluripotent Stem Cell Applications—Induced Pluripotent Stem Cells

Most of the pluripotent lines that that have been investigated by FTIR microspectroscopy have been of embryonic origin, with the extension of this approach to autologous cell (somatic cell derived) induced pluripotent stem cells only presented in the past year. The first group to publish in this area employed synchrotron FTIR microspectroscopy to analyse the FTIR spectra of several ES and iPSC lines. In their first paper, the authors reported that spectra acquired from six ES lines and six unrelated iPSC cell lines were biochemically similar, as indicated by their spectral clusters co-localising in the scores plot, and further confirmed by the PLS-DA prediction modelling data [35]. This result is in agreement with findings from studies performed by our laboratory [36], in which we found marked biochemical differences within the same stem cell class, but no significant interclass spectral variation.

Sandt and colleagues [35] noticed molecular differences between the infrared spectra of the parental somatic amniotic fluid cells (AFC) and their iPSC derivatives, indicated by their spectral clusters segregating along PC1 of the PCA scores plot. The PC loadings, which best described the spectral variance causing the segregation were the bands assigned to the lipids (1740 cm^−1^, 1710 cm^−1^, 1465 cm^−1^, 1455 cm^−1^, 1170 cm^−1^), the proteome (1650 cm^−1^, 1635 cm^−1^, 1550 cm^−1^), and phosphorylation (1270 cm^−1^, 1074 cm^−1^). These biochemical disparities were confirmed by PLS-DA modeling which could successfully discriminate spectra from both stem classes with a 100% accurate prediction. Similarly, spectral differences could also be seen between the murine iPSC cell line (M2A1) and the murine embryonic fibroblast cells (MEF) used in their generation. Again, their macromolecular dissimilarities could be seen by the separation of scores in the PCA scores plot, and by the excellent classification by PLS-DA modelling.

In a further comparison, the FTIR spectra of ESC-H9 mesenchymal stem cells generated from H9 cells (MSC-H9) and iPS cells derived from the MSC-H9 cells (iPSC-H9) were all analysed via multivariate analysis where it was found that the MSC-H9 spectra clustered away from spectra of the colocalising ESC-H9 and iPSC-H9 lines due to the former having higher lipid to protein and lower nucleic acid to protein ratios. Whilst a PLS-DA model using the first 3 PCs explained 85% of the spectral variance could not discriminate the two co-localising stem cell types from each other, a PLS-DA model using 8 PC factors, explaining 99% of the spectral variance was able to discriminate between the two co-localising stem cell types with high accuracy.

However, the most noteworthy finding from the study was the demonstration that the technique was also successful in discriminating fully programmed iPSC lines from partially reprogrammed iPSC lines, with PCA analysis showing a separation of scores pertaining to cells of the two stem cell variants along PC2 and explained by bands in loadings plots associated with increased lipid and glycogen storage in the iPSCs-PR.

Subsequent PLS-DA modelling capturing 98% of the variance (10 factors) was then able to correctly classify the iPSC-FR spectra from the iPSC-PR spectra with 100% sensitivity and specificity. Additionally, biochemical variability was observed in the spectral regions ascribed to protein, glycogen, lipid and nucleic acids between the two cell types at different stages of reprogramming. Nevertheless, the problem with utilising such a large number of factors is that features such as noise or spectral regions that are not of interest to the investigation but are cross correlated may be included in the prediction, thus producing an over-fitted model and reducing its robustness. This was demonstrated by researchers who upon trying to predict glucose features in their fermentation samples, found that other analytes were being included in the predictive analyses when they used more than three PCs in spectral models [37].

**Figure 6:**
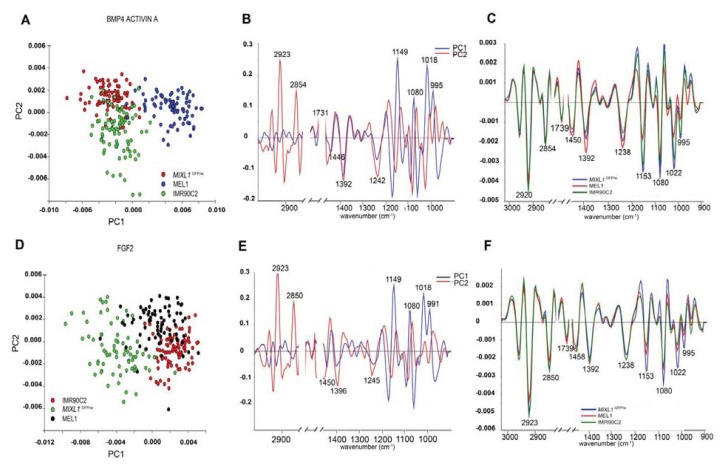
PCA scores plot (**A**) loadings plot (**B**) and average second derivative spectra plot (**C**) for spectra from the hESC lines *MIXL1*^GFP/w^, MEL1 and the hIPSC line IMR90C2 treated with BMP4/ACTIVIN A acquired using FPA-FTIR microspectroscopy. PCA scores plot (**D**) loadings plot (**E**) and average second derivative spectra plot with the highest standard deviation across all of the spectra displayed (**F**) for spectra from the hESC lines *MIXL1*^GFP/w^ and MEL1and the hIPSC line IMR90C2 treated with FGF2 acquired using FPA-FTIR microspectroscopy [36].

A key difference between the studies performed by Sandt and colleagues, and our laboratory is that the former does not address the consequences of differentiation on these spectral phenotypes [32,36]. We already knew that hESC undergoing differentiation towards ectodermal and mesendodermal lineages underwent lipid depletion and were therefore curious to see whether this was also the case with differentiated hIPSC lines, which indeed it was. The surprising finding, however, was that despite the observation that both stem cell variants underwent the same macromolecular transitions during the 4 days of differentiation, the resulting cells appeared to be spectroscopically distinguishable from each other, in spite of the flow cytometry data suggesting that the two stem cell variants had similar differentiation kinetics [36] (Figure 6).

It is feasible that the aforementioned disparities between the two cohorts may reflect different responses to cytokine cues, resulting in lineage committed progeny that appear morphologically equivalent, but are still distinct in terms of their macromolecular composition.

Sandt *et al*. [38] compared the IR spectral profiles of two amniocyte derived IPSC lines that had been generated using different reprogramming. The iPSC line PB09 was transduced with four VSVG-pseudotyped lentiviruses carrying Oct4, Sox2, Lin28 and Nanog (OSLN) vectors. The iPSC line PB10 was, however, transduced using only two exogenous transcription factors, Oct4 and Sox2. Whilst the mean spectra of both these cell lines appeared strikingly different, the groups could not be separated via the first two principal components along a PCA scores plot, although intra-class separation was observed within the PB10 line, along PC 3. Further, PLS-DA modelling of spectra from the two lines showed poor prediction results, with low sensitivity or specificity. From these data, the group concluded that the use of two transcription factors appeared to be sufficient for the generation of biochemically, and thereby, spectroscopically identical iPS cells.

Whilst these findings are interesting, the authors’ claims would have been strengthened by the investigation of a greater number of cell lines. Another suggestion for follow up studies would be to determine whether these observed biochemical similarities persisted upon differentiation.

## 7. Multipotent Stem Cell Applications

### 7.1. Mesenchymal Stem Cells

FTIR microspectroscopy has been applied to a varied range of adult stem cell types in multipotent stem cell studies. Krafft and colleagues were the first group to probe human mesenchymal stem cell (hMSC) differentiation at the single cell level [39]. In their work, the IR spectra of several hundred single human mesenchymal stem cells with or without osteogenic stimulation were recorded, with the non-stimulated control cells consisting mainly of populations that had either high or low levels of peripheral glycogen levels, whereas stimulated cells showed alterations in protein composition and expression of octacalcium phosphate, a calcium phosphate salt. However, the main discriminants between the non-stimulated and stimulated classes were attributed to protein differences. For instance, spectra of the non-stimulated hMSCs consisted of a more intense peak at 3285 cm^−1^ and at 1631 cm^−1^, the spectral regions which were assigned to the amide A and β-sheet protein secondary structure, respectively. A criticism of this study is that absorbance at the amide A band 3285 cm^−1^ band can also arise from the O-H stretching mode of water and since this wasn’t accounted for, it is feasible that differences in this peak intensity could be partially ascribed to differences in the hydration state of the samples.

The spectroscopic technique has also been adopted for studies of rat bone marrow mesenchymal stem cells (rBM-MSCs) differentiated to hepatocytes [27]. The main findings from the work were that the late stage differentiated cells possessed significantly higher levels of lipid compared to the stem cell progenitors and early stage differentiated cells, with notable differences in the FTIR absorbance bands at: 3012 cm^−1^ (cis C=C stretch from unsaturated lipids), 2952 cm^−1^ (ν_as_CH_3_ from lipids), 2854 cm^−1^ (ν_s_CH_2_ from lipids) and 1722 cm^−1^ (C=O stretching from lipids). The observed increase in lipid content during hepatocyte differentiation differed from what was found in previously described work by Thumanu *et al*. [34] who studied the hepatogenesis of mouse embryonic stem cells and observed higher lipid levels in the progenitor cells. These disparities could have been attributed to differences in lipid signaling between the two stem cells types. Further work that would provide a greater insight into these findings could involve inducing hepatogenesis in both stem cell variants in parallel and then doing a direct comparison of the hepatocyte-like cells derived from embryonic, and MSCs, to each other.

The results from Ye *et al*. [27] aligned with non-spectroscopic studies which report that hepatocytes routinely synthesise lipoproteins and synthesise and store triglycerides as a result of activities from carbohydrate metabolic pathways. Moreover, a significant increase in unsaturated fatty acid levels was seen in the late stage hepatocytes, indicated by an increase in the intensity of C–H stretching band associated with the cis double bond C=C at 3010 cm^−1^. However, changes in band intensity in spectral regions representing proteins and nucleic acids were more complex and therefore more difficult to interpret compared to the studies carried out by Thumanu and colleagues.

In another study investigating this stem cell type, Chonanant *et al*. [40] employed synchrotron FTIR microspectroscopy for the first time to characterise the chondrogenic differentiation of human mesenchymal cells generated via the pellet culture method with and without stimulation by growth factors (TGF-β3 and BMP-6), over a period of 7, 14 and 21 days. Inspection of the average second derivative spectra revealed that the chondrocyte induced hMSCs were characterised as having higher absorbance at 1338 cm^−1^, 1230 cm^−1^ and 1203 cm^−1^, assigned to the amide III, P–O stretching and C–O–C stretching modes of collagen type II, respectively. Further, spectra of the chondrocyte induced group had higher absorbance at 1152 cm^−1^, 1107 cm^−1^, 1080 cm^−1^, 1019 cm^−1^, and 993 cm^−1^, attributed to aggrecans which were higher in chondrocyte-induced hMSCs than in the controls. The ability to detect chondrogenic differentiation was found to be high, with a clear separation of spectral clusters in scores plots and the regression loadings revealing differences that followed a similar pattern to what was observed in the average second derivative spectra. PLS-DA using the independent test spectra showed that the two spectral classes could be discriminated from each other with 100% accuracy.

Considering the high heterogeneity of pellet culture differentiated cells, compared to those derived from monolayer-based protocols, the exploration of the latter method in future work would be a worthy pursuit. Moreover, the researchers who examined paraffin sections need to be mindful that biochemical changes can arise from tissue processing, which will affect the spectral signatures of the sample. This was shown in experiments by Faolain and colleagues who saw shifts of 10 cm^−1^ in the amide I and II bands, ascribed to the cross-linking of proteins, caused by formalin fixation [41]. The authors advised against the diagnostic use of biological bands that are close to fixative peaks because even after careful washing, trace remnants may still persist.

### 7.2. Corneal Stem Cells

SR-FTIR microspectroscopy was undertaken by one research group in their experiments using bovine corneal epithelium [9]. The cell types that they investigated were the lifelong proliferating putative adult stem cells (SC), the SC derived progenitor or transiently amplified (TA) cells which have only limited proliferative capacities, and the TA-derived, non-proliferative terminally differentiated (TD) cell populations. PCA of the spectra acquired from the corneal sections, showed the distinct clustering of spectra from the three different cell types, with only a slight overlapping of the spectral clusters of the SC and TA groups, possibly due to the presence of a small population of TA cells that had yet to migrate out of SC niche at the time. Statistically significant spectral differences (*p* < 0.001), as determined by the Mann Whitney test, were found to occur at the following peaks: 1714 cm^−1^, 1600 cm^−1^, 1450 cm^−1^ to 1440 cm^−1^ and 1225 cm^−1^, all of which are known to be associated with nucleic acids. The spectra of the TD cells, whilst appearing distinctly clustered away from the other spectral groups in the PCA scores plot, were found to be more spectrally aligned with the TA cells than to the SCs, although the TA and TD spectra showed marked dissimilarities, mainly in the regions assigned to protein and RNA. All of the observed differences were thought to be related to changes in chromatin structure, which is known to be linked to cell differentiation.

SR-FTIR microspectroscopy combined with spectral imaging has also been used successfully for the characterization and localisation of the biomarkers of SCs, TA cells, and TD cells in human derived corneal sections [42]. PCA of the single point spectra acquired from the three putative cell type regions (SC *vs.* TA cell *vs.* TD cell) revealed an excellent separation between the spectral classes. Wavenumbers that were found to be differentially absorbed by the SC classes compared to the other two cell types were ascribed to DNA (1040 cm^−1^, 1080 cm^−1^, 1107 cm^−1^ and 1225 cm^−1^), with some contributions from C–O stretching in carbohydrates derived from amino acid side chains or lipids at 1400 cm^−1^, amide II absorption bands at 1525 cm^−1^ and 1558 cm^−1^, and the lipid associated band at 1728 cm^−1^. Proteins and lipids were the entities that most distinguished TA cells and TD cells. These two studies demonstrate the utility of SR-FTIR microspectroscopy to discriminate and segregate putative populations of SCs, TA and TD cells of the bovine and human corneal epithelium.

### 7.3. Gastrointestinal Crypt Stem Cells

This spectroscopic approach has proved useful for studying the complex stem cell niche of the small and large human intestine. Both globar and synchrotron based FTIR microspectroscopy were employed by a leading group in an attempt to segregate and characterise the putative stem cells, transit amplifying cells and differentiated cells of human intestinal crypts [43]. These cells reside in different regions along the length of the gut, with locations differing between the small intestine, and large intestine originated crypts. PCA-LDA of spectra obtained from the aforementioned cell types showed a clear segregation of scores related to the various classes. The most prominent separation of scores was attributed to the spectral region assigned to DNA/RNA, with the symmetric PO_2_^−^ (1080 cm^−1^) vibrations found to be a marker for the putative stem cell region possibly due to changes in chromatin. These same biomolecular signature differences between the different stem cell classes were found to be similar regardless of whether the cells were derived from small intestine or large intestinal crypts.

**Figure 7:**
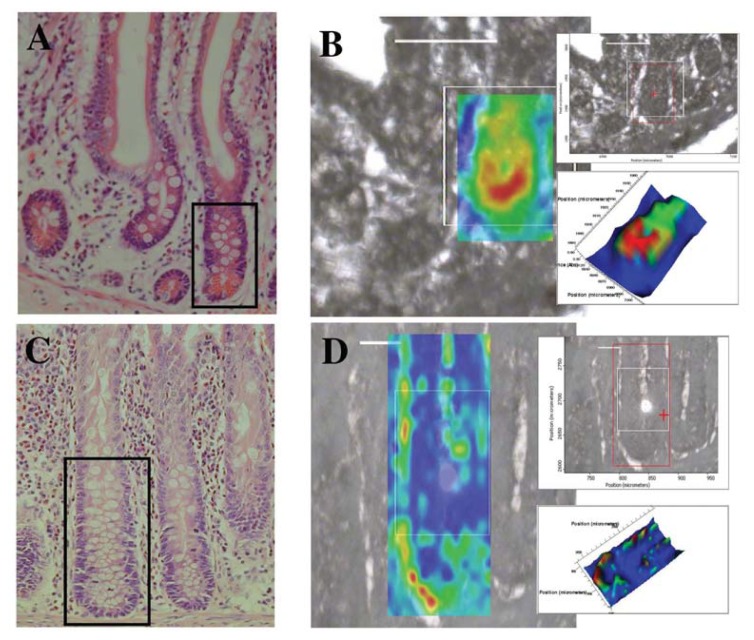
Localization of the putative stem cell region in an IR spectral image (resolution = 10 μm × 10 μm) map of human GI crypts using synchrotron FTIR microspectroscopy. (**A**) A section of small intestine tissue stained with H & E post-interrogation with synchrotron FTIR microspectroscopy—the imaged area is designated by the black box; (**B**) a two-dimensional map of a small intestine crypt, smoothed at wavenumber 1080 cm^−1^ and superimposed on the unstained region analyzed—see inset. (**C**) A section of large bowel tissue stained with H & E post-interrogation with synchrotron FTIR microspectroscopy—the imaged area is designated by the black box. and (**D**), a two-dimensional map of a large bowel crypt, smoothed at wavenumber 1080 cm^−^1 and superimposed on the unstained region analyzed—see inset. Absorbance intensity (see insets) is proportional to thermal colour changes: blue (lowest intensity) < green < yellow < red (highest intensity) [8].

The same authors in another study used synchrotron FTIR microscopy to generate IR image maps of small intestinal and large intestinal crypts [8] (Figure 7). The cell types that were interrogated were assigned step-wise positions along the length of the crypt. In the small intestine, the cell types and their positions were as follows: cells residing in the crypt base (Position 1), crypt base columnar (CBC)/Paneth cells (Position 2,3), label retaining cells (LRCs) (Position 4–6), transit amplifying (TA) cells (Position 7,8), and terminally differentiated (TD) cells (Position 9,10). The putative cell types and assigned positions that were probed in the large intestine crypts were the putative stem cells (Position 1–4), TA cells (Position 5–8), and TD cells (Position 9,10). PCA-LDA of spectra derived from both small intestine and large intestine crypts found that the spectral regions that most contributed to the variance segregating different putative cell types occurred at 970 cm^−1^ (protein and nucleic acid phosphorylation), ν_s_PO_2_^−^ (1080 cm^−1^) and ν_as_PO_2_^−^ (1225 cm^−1^).

Analysis of individual and combined spectra acquired from the small intestine-derived crypts showed that the greatest biochemical differences were between the spectra of the cells that resided in Position 1, and TD cells, and that the base of small intestine crypts was the most spectrally distinct region. Spectra from the intermingling CBC and Paneth cells were found to be spectroscopically disparate from the spectra of cells that resided in Position 1, whereas they shared biochemical similarities with LRCs and TA cells in spectral regions pertaining to DNA/RNA and protein, with the latter region causing the most prominent separation. However in the case of the spectra derived from large intestine crypts, the greatest segregation of spectral clusters occurred between the putative stem cells located in Positions 1–3 away from the closely clustered TA and TD spectra. Conversely, putative stem cells derived from Position 4 often clustered closely with TA derived spectra.

Upon comparing the spectra of these cell types from both the small and large intestine crypts, it was observed that relative to the spectra of the CBC/Paneth cells, the label-retaining cells were spectroscopically similar to the large bowel derived putative stem cells. The small intestine derived spectra of the TA cells were spectroscopically similar to the spectra of the large intestine-derived TA cells. Interestingly, inspection of the PCA scores plot showed the Paneth spectra clustering between the clusters of LRC and TA clusters, suggesting that these cells possessed a biochemical “fingerprint” that was intermediate between these two putative cell types.

### 7.4. Other Applications of FTIR Microspectroscopy to Stem Cell Research

#### 7.4.1. Haematopoietic Stem Cells

Globar FTIR microspectroscopy has the capacity to discriminate isolated murine haematopoietic stem cells (HSCs) from bone marrow (BM) cells [44]. PCA of spectra derived from these cells, revealed that they could be distinguished from each other as evidenced by the separation of their spectral clusters along PC1, with the biochemical entity which most contributed to the observed variance being DNA, assigned to the loadings bands at 966 cm^−1^, 1088 cm^−1^ and 1240 cm^−1^. These bands were found to have higher IR intensities in the HSCs compared to the BM cells, possibly due to the former having a unique chromatin conformation. HSCs could also be identified from BM cells by having higher lipid absorbance, suggestive of the stem cells having a unique membrane composition and structure. When determining the utility of this methodology for quantifying HSCs in the bone marrow by using the characteristic wavenumber biomarkers of HSC previously identified to be associated with DNA, the group found that one HSC per thousand BM cells could be successfully identified.

#### 7.4.2. Cancer Stem Cells

The lack of specific cancer stem cell biomarkers for the isolation of putative stem-like cells from either normal or malignant renal tissue, led to exploration of the ability of high brilliance synchrotron-FTIR spectroscopy to discern the unique spectral markers of three isolated cell types residing in this tissue [26]. The extraction of these cell types was achieved using the Hoechst 33,342 dye efflux assay, from which the 3 sub-populations that were used for analysis were the non-SP (side population) containing differentiated cells and 2 SP (sub-populations); the proximal side population (PSP); and the distal side population (DSP). Of these groups, the latter two subtypes were known to consist of putative transit amplifying cells, and the most primitive (stem) cell types respectively. PCA of spectra acquired from the different cell types revealed a clear segregation of scores pertaining to the Non-SP group away from the DSP and PSP group, with the key biochemical differences explaining the separation being changes in lipids, phosphodiester groups and carbohydrates. Principal component–linear discriminant analysis (PC–LDA) revealed that the independent validation DSP, PSP and Non-SP dataset had at least 80% of their spectra correctly assigned.

When pairwise PCA comparisons were then performed using only the spectral datasets of the DSP and Non-DSP groups, a segregation of scores from the two cell types was witnessed, with spectra from all cell subtypes from the DSP group found to be more spectrally homogeneous, tightly clustering together compared to the more spectrally heterogeneous spectra from the Non-SP groups, which were found to form two groups. The main biochemical moieties causing this spectral discrimination were the lipid components, denoted by loadings bands at 1468 cm^−1^ and at 1380 cm^−1^. Along PC2, the PC where the non-SP spectra segregate into two clusters, the loadings bands were dominated by carbohydrates and symmetric and asymmetric phosphodiester signatures. Finally, the group compared the spectral datasets of the DSP and the PSP cells and found a clear separation between the two cell types along PC1 due to differences in the phosphodiester stretching band vibrations. By using FTIR microspectroscopy, the group established that the lipid and phosphodiester vibrations were the most important markers for discriminating stem cell-like cells from the more differentiated variants.

### 7.5. Unique Macromolecular Chemical Signature Differences Exist between Undifferentiated and Differentiated Stem Cells

A common observation in both of the pluripotent and multipotent stem cell studies was that stem cells undergoing differentiation possessed different lipid “signatures” from their lineage committed progeny. However, there has been no consensus on a “lipid profile” that is common to all stem cell types, although the possible effect of culturing environment on the these spectral profiles cannot be dismissed. For instance, in the hepatocyte differentiation studies performed by Thumanu *et al*. [34], and human embryonic stem cell differentiation work carried out by Heraud *et al*. [32], lipid depletion was found to co-occur with a loss of pluripotency. However, the contrary was found to be the case by Tanthanuch *et al*. [29] in their study of mouse embryonic stem cell derived neuronal cells, and by Ami *et al*. [33], who did not report any significant lipid differences between their undifferentiated and spontaneously murine differentiated stem cells, but instead noticed differences in protein and nucleic acid spectral signatures between the stem cells and their derived cell types.

The interpretation of protein information, especially when the amide I band is involved, needs to be done carefully due to spectral artefacts such as resonant Mie-scattering being particularly prominent in this spectral region. For example, Thumanu *et al*. [34] were able to verify that the observed amide I band changes seen in their study were actually protein related, rather than artefactual, by noting that these alterations also coincided with the appearance of new band features, rather than simple wavenumber shifts in the second derivative spectra. Further, the authors observed that these protein changes coincided with albumin formation, a protein alteration known to occur during the differentiation of hepatocytes.

Care must also be taken when interpreting changes in the nucleic acid region given that the intensity of these band features are affected by different levels of hydration. An example of this could be seen in work by Whelan *et al*. [45] who showed that DNA specific bands in several eukaryotic cells were more intense in hydrated samples compared with dehydrated samples.

### 7.6. FTIR Spectroscopic Signatures of Stem Cells are Influenced by the Growth Environment

In light of the findings that FTIR microspectroscopy can be used to classify pluripotent and multipotent stem cells based on their spectral phenotype, there are preliminary studies addressing the question of how the environment might affect the results. The genotypic and phenotypic consequences of using different culturing conditions for the maintenance and differentiation of stem cells has been well established by non-spectroscopic methods [46–48]. By contrast, the influences of the growth milieu on stem cell spectral biomarkers have been the subject of only two studies to date.

Pijanka *et al*. [31], explored this question by testing the effect of two different oxygen concentrations (2% and 21%) on the FTIR spectroscopic signatures of hESC and hMSC using synchrotron radiation FTIR microspectroscopy. The group found spectroscopic differences between the hESC and hMSC spectra when cultured in either oxygen concentration, with the former displaying more intense peaks in spectral regions assigned to lipid components (2850 cm^−1^, 2920 cm^−1^ and 1740 cm^−1^). The findings that the spectra from the more differentiated hMSCs possessed a lower lipid content than the less differentiated hESC spectra, is in agreement with data reported by our colleagues, and by others. At present, the functional significance of these lipid differences is not yet known although it has been speculated that the eicosanoid pathway may play a role [49]. However, the observation of higher lipid levels in more differentiated stem cells by other groups suggests that this may be a cell-type specific phenomenon and will warrant further investigation.

**Figure 8:**
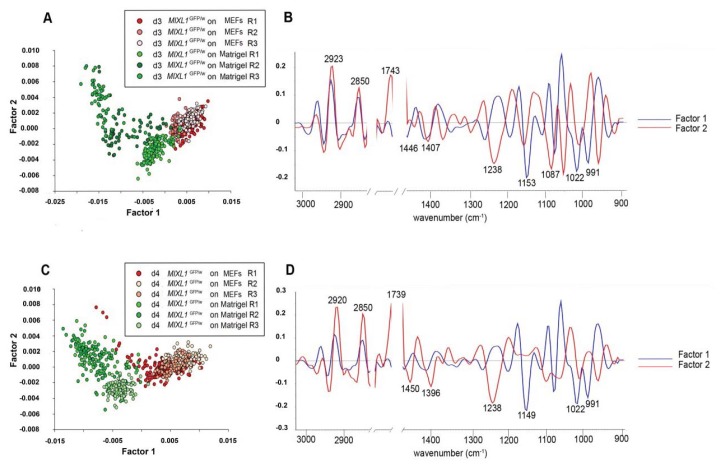
PLS-DA scores plots (**A**), and loadings plots (**B**) of spectra acquired from *MIXL1*^GFP/w^ after 3 days of differentiation towards mesendodermal lineages using BMP4 ACTIVIN A. PLS-DA scores plots (**C**), and loadings plots (**D**) of spectra acquired from *MIXL1*^GFP/w^ after 4 days of differentiation towards mesendodermal lineages [50].

Our laboratory also examined the effects of the cell culture environment on the spectroscopic phenotype of human embryonic stem cells by analysing the spectral profiles of various human embryonic stem cell lines maintained under three different cell culture systems [50]. The study yielded several noteworthy findings. For instance, it was discovered that hESC lines maintained in bulk culture, organ culture, and the commercial synthetic substrate Matrigel all possessed distinctly different FTIR spectroscopic signatures due to the influence of both the growth medium and the growth matrix. Further, these original culture conditions were found to have a continuing effect on the stem cells’ spectral “signatures”, even after several passages onto a different tissue culture platform, and even upon differentiation towards a defined lineage commitment state. For instance, spectroscopic differences still prevailed after 4 days of differentiation towards a mesendodermal lineage between cells derived from progenitor *MIXL1*^GFP/w^ cells maintained in bulk culture, and those maintained on Matrigel, in spite of the flow cytometry data showing that both groups had highly comparable differentiation patterns and kinetics (Figure 8).

### 7.7. The Importance of Correlative Methodologies for the Interpretation of Stem Cell Spectral Signatures

To facilitate the interpretation of spectroscopic data, particularly when differences reside in spectral regions containing band contributions from multiple macromolecular classes, it is critical to correlate these findings with those derived from non-spectroscopic methodologies. Common techniques that have been used in conjunction with FTIR microspectroscopy stem cell studies have included morphological analysis, flow cytometry, gene expression analysis, fluorescence studies, functional colony assays and histological staining. Despite its popular adoption in stem cell spectroscopy work, genotypic methods such as gene chip analysis may not be directly comparable to findings derived from spectroscopy, since the latter, being a phenotypic technique, is best compared to data derived from other phenotypic protocols. For example, a problem of using gene chip analysis, as was demonstrated in a study by our laboratory [32], is that the data may be uncoupled from the spectroscopic information due to the occurrence of events such as post-transcriptional modifications.

## 8. Future Directions

Although the application of Fourier transform infrared spectroscopy to the study of pluripotent and multipotent stem cells is still in its infancy, it has already been successfully demonstrated with different murine and human stem cells and their various differentiated cell types. However, before the benefits of spectroscopy in stem cell research can be fully realised, several important developments must be made. For example, advances in instrumentation to speed up data acquisition and improve signal to noise ratios would help to significantly further the field. The most recent progress in this area, as described earlier, has been the coupling of FPA detectors to synchrotron light sources, and the use of quantum cascade laser sources of infrared radiation. In addition to instrumental development, there is a critical and growing need for a focus on metrology to further the diagnostic potential of FTIR microspectroscopy. Standardising measurement parameters such as spectral resolution, the number of co-added scans, and the number of binned pixels will allow for results between studies to be more readily comparable. In this context, instrument inter-comparisons are necessary to gauge the effects of instrument type on spectral classification. Also imperative is a better understanding of the effects of confounding variables on spectroscopic data and ways to minimise these or to correct for the resulting spectral artefacts by data pre-processing.

Due to its ability to provide objective phenotypic information in a rapid and non-invasive manner, complementary to those derived from more conventional modalities, we anticipate the continuing use of infrared microspectroscopy in future stem cell research.

## Figures and Tables

**Table 1:** Band assignments of mid-IR spectra common to biological samples.

Band maxima (cm^−1^)	Band assignments
~2962	C–H stretching peak of lipids and proteins
~2923	C–H asymmetrical stretching of lipid groups and protein
~2850	C–H symmetrical stretching of lipid groups
~1743	C=O stretching of lipid esters
~1685	β-turn protein secondary structure
~1654	α-helical protein secondary structure
~1635	β-pleated sheet protein secondary structure
~1554	Overall protein absorbance
~1458	Methyl and methylene groups from lipids and protein
~1396	COO^−^ stretching vibrations of amino acid side chains
~1238	P=O asymmetrical stretching of PO_2_ phosphodiester groups from phosphorylated molecules
~1080	P=O symmetrical stretching of PO_2_ phosphodiester groups from phosphorylated molecules, and glycogen
~1153	C–O vibrations from glycogen and other carbohydrates
~1050	C–O vibrations from glycogen and other carbohydrates
~1022	C–O vibrations from glycogen and other carbohydrates
~995	C–O stretch from RNA ribose chain and other carbohydrates
~950	C–C vibrations from nucleic acids
